# Abdominal functional electrical stimulation and binder to reduce orthostatic hypotension in individuals with a subacute spinal cord injury

**DOI:** 10.1038/s41394-026-00757-x

**Published:** 2026-10-19

**Authors:** Chiettha Prajnadewie, Lynn Blecher, Keith MD McNaughton, Claire L. Boswell-Ruys, Billy L. Luu, Harrison T. Finn, Elizabeth A. Bye, Bonsan B. Lee, Jane E. Butler, Anne E. Palermo, Euan J. McCaughey

**Affiliations:** 1 University of New South Wales, Sydney, NSW Australia; 2 Neuroscience Research Australia, Sydney, NSW Australia; 3 The Kolling Institute and School of Medical Sciences, Faculty of Medicine and Health, The University of Sydney, Sydney, NSW Australia; 4 Prince of Wales Hospital, Sydney, NSW Australia; 5 TIRR, Memorial Hermann, Houston, USA; 6 Queen Elizabeth National Spinal Injuries Unit, Queen Elizabeth University Hospital, Glasgow, Scotland G514TF UK; 7 College of Medicine, Veterinary and Life Sciences, University of Glasgow, Glasgow, Scotland G128QQ UK

**Keywords:** Medical research, Neuroscience, Neurophysiology

## Abstract

**Study design:**

Repeated measures comparative study.

**Objectives:**

Compare acute effects of 4 treatment conditions: (i) abdominal binder (Binder), (ii) abdominal functional electrical stimulation (AFES), (iii) AFES and binder (AFES-Binder), and (iv) no intervention (Control), on signs of orthostatic hypotension (OH) and blood pressure (BP) maintenance in individuals with acute and subacute spinal cord injury (SCI).

**Setting:**

Spinal injury inpatient ward.

**Methods:**

Eleven participants with SCI (C3-T5) with at least one sign or symptom of OH underwent a passive sit-up test on 4 days with the 4 treatment conditions. Non-invasive continuous recordings of systolic BP, diastolic BP and calculated mean arterial pressure (MAP) were measured during 4 periods: (1) supine without treatment (5 min); (2) supine with treatment (5 min); (3) upright with treatment (up to 15 min); and (4) supine post-treatment (10 min).

**Results:**

Largest drops in BP still resulted in OH in all conditions (*p* < 0.001), with the largest instantaneous change in MAP in the Binder condition (−41 mmHg [95% CI, −47 to −34]). AFES-Binder condition maintained mean upright MAP relative to supine (−1 mmHg [95% CI, −5 to 2], p = 0.291). Mean upright MAP decreased in all other conditions [Control: (−4 mmHg [95% CI, −7 to −1], p = 0.002); Binder: (−7 mmHg [−10 to −4], *p* < 0.001); AFES: (−6 mmHg [95% CI, −9 to −2], *p* < 0.001)]. Mean upright MAP was highest in the AFES-Binder condition.

**Conclusions:**

No single treatment condition prevented OH completely. While AFES with abdominal binder does not eliminate OH, it shows potential to mitigate BP instability during postural transitions in acute and subacute SCI.

## Introduction

A spinal cord injury (SCI) at T6 and above can alter autonomic control and lead to blood pressure (BP) dysregulation and orthostatic hypotension (OH). Vasoconstriction is impaired after SCI, which causes poor venous return and blood to pool in the abdominal cavity and lower extremities [1]. OH, defined as a drop of ≥20 mmHg in systolic or ≥10 mmHg in diastolic BP from supine to the upright position, is common during the acute phase of injury and affects over 50% of individuals living with a SCI [2, 3]. OH impedes early participation in rehabilitation and accounts for over 30% of ceased therapy sessions in people with acute SCI [2]. OH encompasses multiple haemodynamic subtypes: initial OH, characterised by a transient drop in BP upon change to the upright position; delayed BP recovery, in which recovery from a drop in BP is slowed; classic OH, defined by a sustained drop in BP within 3 min in the upright position; and delayed OH, in which a sustained drop in BP occurs after 3 min [4]. In addition, upright BP is lower in individuals with cervical SCI compared to individuals with thoracic SCI and neurologically intact individuals [5], which may affect their ability to tolerate and function in the upright position over time.

Recommendations for the management of OH post-SCI prioritise individualised approaches that focus on corrective behaviours and non-pharmacological strategies [4]. Corrective strategies include ensuring adequate hydration and physical counterpressure manoeuvres but these alone may not be sufficient [4, 6]. Non-pharmacological strategies involve compression garments such as abdominal binders and compression stockings, which aim to promote venous return through physical constriction [7]. However, while abdominal binders are part of standard rehabilitation care, there is limited evidence of their effectiveness on OH and BP maintenance in acute SCI [8]. Meanwhile, pharmacological therapies like midodrine are effective in elevating BP through vasoconstriction [9], but should only be a secondary resort as they are not position-dependent and may cause the adverse effect of supine hypertension [10].

Functional electrical stimulation (FES) activates weak or paralysed muscles through the delivery of electrical currents over the nerves which innervate the muscles and can aid in BP management [3]. FES that targets abdominal muscles, or abdominal FES (AFES), may create a muscle pump that limits the stasis of blood in the abdominal cavity. Furthermore, stimulation of sensory afferents with AFES may increase sympathetic outflow and increase BP [3]. Most investigations of AFES on BP regulation in SCI are either limited to a case study [11], secondary observations when targeting other outcomes [12, 13], or do not consider postural changes in their methods [13]. In these studies, the administration of AFES resulted in significant increases in systolic BP but had transient periods of efficacy, renewable with repeated bouts in one case [11], yet diminishing over time in another [13]. Currently, only one study has investigated AFES as a management strategy for OH in individuals with subacute SCI [14], but it did not examine the abdominal binder. Instead, it compared AFES to quadriceps electrical stimulation and compression stockings [14]. Given the binder’s routine use in clinical care, a powered evaluation of non-pharmacological therapeutic options for OH post-SCI, including its use, is required to optimise clinical management and improve participation in therapy.

The current study compares the effects of no intervention (Control), the abdominal binder (Binder), AFES, and combined AFES and abdominal binder (AFES-Binder) on BP during orthostatic challenges in people with a subacute SCI. While OH focuses on minimum upright BP that captures transient lows which may trigger symptoms, mean upright BP reflects sustained perfusion that provides insight into the degree of autonomic impairment. An improved understanding of both can help elucidate the functional impacts of autonomic dysfunction in SCI [5]. By comparing BP across conditions, we aimed to (i) identify whether the treatment interventions could prevent orthostatic drops in BP in the upright position and (ii) investigate how the conditions affected the overall maintenance of upright BP. We hypothesised that both the AFES and AFES-Binder conditions would independently reduce orthostatic drops in BP and maintain upright BP.

## Methods

### Study design

In this randomised crossover repeated measures study, each participant completed four sessions on separate days within a two-week period. One of the four treatment conditions (Control, Binder, AFES, and AFES-Binder) was used per session. Assessments were conducted between 6:00am to 8:30am for 10 of 11 participants to align with the time of day when OH is typically most pronounced in acute SCI [2, 15]. In the remaining participant, assessments were conducted later in the afternoon between 3 pm to 4:30 pm to align with the participant’s schedule, except for one session which was conducted in the morning between 7:00am to 8:30am.

### Participants

Individuals were eligible for study participation if they were ≥18 years old, had a recent (<1-year) SCI with a neurological level between C1-T5, American Spinal Injury Association Impairment Scale A-D, cleared to sit, and a sign or symptom of OH. Individuals were excluded if they had progressive neurological disease, no response to AFES, physical obstacles or contraindications to AFES, cognitive or language barrier, or if there was active titration of BP-altering medications. They were also excluded if they were undergoing active treatment for a known infection at the time of recruitment, and if their morning BP was consistently >140 mmHg in the medical records. All participants provided informed consent. This study protocol was approved by the South Eastern Sydney Local Health District Human Research Ethics Committee (Ethics no. 2019/ETH13271) and conformed with the Declaration of Helsinki (2013), except for prior registration in a public database. An a priori power analysis was conducted based on means and standard deviations (SD) from McBain et al. [12]. A sample size of 12 accounted for an attrition rate of 30% with a significance level set to < 0.05 and resulted in a power of 0.97.

### Abdominal binders and AFES

**Fig. 1:**
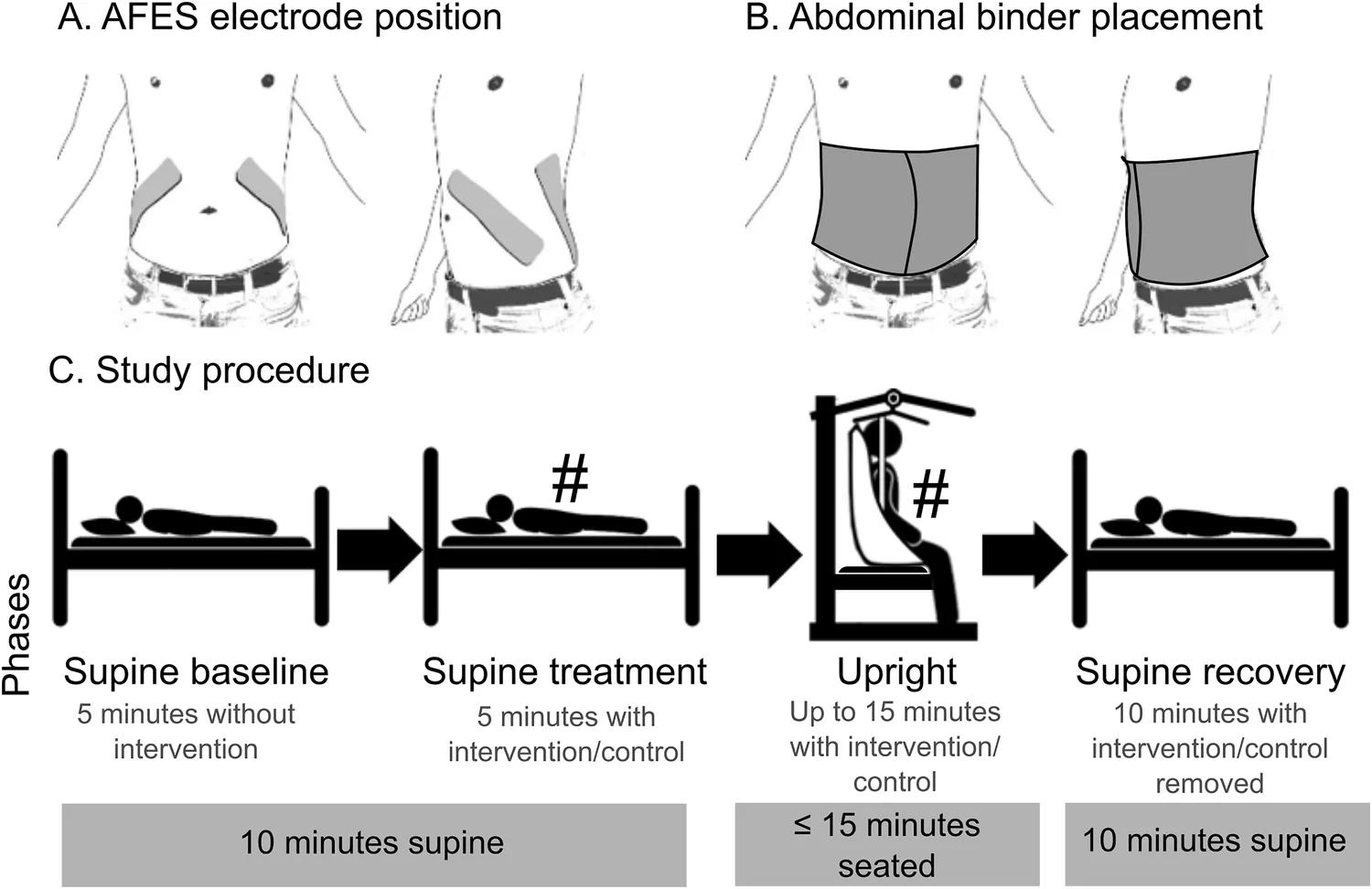
Study procedure and AFES electrode position. Participants completed an orthostatic challenge on four separate days with a different treatment condition: no intervention, binder, abdominal functional electrical stimulation (AFES), and AFES and binder combined, in a randomised order. **A** For conditions where AFES was used, 4 surface electrodes were positioned over the skin along the medial and lateral abdomen. Electrode placement: two anterolateral electrodes were placed in the space that spanned from 2 cm below the costal margin across to the anterior superior iliac spine, and 2 posterolateral electrodes were placed from the level of T8 vertebra on the mid-axillary line towards the posterior superior iliac spine. These electrodes delivered electrical currents transcutaneously to the nerves innervating the abdominal muscles to initiate a contraction during exhalation. Image adapted with permission from McCaughey et al. (2018). **B** For conditions where the abdominal binder was used, the binder was applied around the lower abdomen to extend from below the costal margin to the anterior superior iliac spine. **C** On each day, blood pressure was recorded continuously over 4 phases: a 5-min supine baseline phase (without treatment condition); 5-min supine treatment phase (with treatment condition), up to 15 min after transition to the upright phase (with treatment condition), and 10 min in the supine recovery phase (with treatment condition removed) post-sit. Participants were seated over the edge of a bed supported in a sling and experienced physiotherapists supported the participant throughout the entirety of the upright phase. ^#^indicates application of treatment condition.

Standard abdominal binders (Thuasne abdominal binder, Thuasne SAS, France) provided to all patients with SCI by the local hospital were used. Binders were donned by experienced physiotherapists. AFES (VentFree^TM^ electrical muscle stimulator, Liberate Medical LLC, USA), triggered to stimulate during expiration as detected by a flow sensor, was delivered via four adhesive electrodes (5 × 15 cm) placed over the posterolateral skin of the abdomen [16] (Fig. 1A). The intensity of the AFES (20–65 mA, 50 Hz, 350 µs pulse width) was titrated to generate a strong, visible, symmetrical and tolerable contraction of the bilateral abdominal muscles.

### Blood pressure measurements

Non-invasive beat-to-beat systolic BP (SBP), diastolic BP (DBP), mean arterial pressure (MAP) and heart rate (HR) were measured using a finger-cuff sensor (Finapres Nova, Finapres Medical Systems, Enschede, Netherlands). All Finapres measures were calibrated via an ipsilateral brachial cuff and height correction unit. To cross-check accuracy of the Finapres recordings, SBP, DBP and HR were measured from the contralateral arm at 5-min intervals with a standard BP monitor (Welch Allyn Connex Spot Monitor, Hill-Rom Holdings, Inc., Chicago, USA). Symptoms of OH were self-reported with the Calgary Presyncope Form. Scores ranged from 0–10 representing an ascending severity of symptoms [17]. Study protocol was ceased and the participant was returned to supine if a score of ≥4 was reported. Signs and symptoms of autonomic dysreflexia were monitored for safety.

### Experimental session: orthostatic challenge

Participants were instructed to remain relaxed and to limit arm movement throughout the following 4 phases of an adapted sit-up test [18]: (i) supine-baseline phase without treatment condition, for 5 min; (ii) supine-treatment phase with treatment condition applied for 5 min to allow the participant to adjust to the treatment; (iii) upright phase with the treatment condition applied for up to 15 min; and, (iv) supine-recovery phase without the treatment condition for 10 min (Fig. 1C). Participants were passively transitioned into the seated, upright position with a sling, placed prior to data collection, and portable ceiling hoist (Molift Group A/S, Matraville, Australia and Winncare Nordic Aps, Korsor, Denmark). Assessments were completed in the bed of the participant on either a fully inflated air or hybrid hospital mattress.

### Analysis and statistics

Data analyses were conducted using CED Spike2 (Cambridge Electronic Design Ltd, Milton, UK), with a custom script that visualised BP and HR against time, and R (version 4.2, The R Foundation for Statistical Computing, Vienna, Austria). The mean of MAP, SBP, DBP and HR were calculated for each phase over one-minute intervals from their respective waveforms. Meanwhile, maximum HR and minimum SBP and DBP corresponded to the single highest and lowest value within each phase, respectively. The last two minutes of the supine-treatment and upright phases were excluded from analysis to limit the impact of preparation for transition between postures on the data. One participant tolerated the upright phase for only two minutes. Here, only the last minute prior to returning to supine was excluded from analysis.

Linear mixed models evaluated fixed effects of “condition” (Control, Binder, AFES, and AFES-Binder) and “phase” (supine-treatment, upright and supine-recovery phases), and their interaction with random effect of “participant” for each variable (MAP, SBP, DBP and HR). Mean values from the supine-baseline phase were used as a co-variate in each model to account for potential within-subject differences in baseline between testing sessions. Minimum SBP and DBP in the upright phase and mean values of MAP, SBP and DBP were compared to the mean values of supine-treatment and supine-recovery phases to assess orthostatic drops in BP and BP maintenance, respectively. An orthostatic drop in BP was calculated from minimum upright BP – mean supine-treatment BP. The primary outcome of the study was the orthostatic drop in SBP. All other outcomes were secondary. The time at which minimum upright BP occurred was recorded as an occurrence and grouped into three bins relative to the start of the upright phase: within 3 min, between 3–10 min, and after 10 min. The means and SD of time at the minimum upright BP and total time in the upright position were also calculated. Presyncope scores were also compared across conditions and across the four phases as an exploratory analysis to assess symptoms.

Data are expressed as means ± SD, medians (interquartile ranges) or estimated marginal means [95% confidence interval]. The outcome variable ‘mean overall’ reflects model estimates derived from fixed effects across all phases, whereas phase-specific outcomes (e.g., ‘mean upright MAP’) represent model estimates restricted to individual phases. Significance was set as < 0.05, with multiple comparisons corrected using Tukey’s method.

## Results

### Participant characteristics

Of 156 patients screened, 16 were eligible for the study. Eleven individuals consented and were enrolled (Table 1). Participant #10 withdrew after one session (no intervention condition) due to recurring morning hypertension (SBP > 140 mmHg). For Participant #8, non-continuous BP measurements were used in the analysis for two sessions when technical issues prevented continuous BP recordings. Therefore, beat-to-beat BP and HR were collected from 11 Control, 10 Binder, 9 AFES and 9 AFES-Binder sessions while non-continuous BP and HR were collected from one AFES and one AFES-Binder session. In one Binder and one AFES session, technical issues upon return to supine resulted in beat-to-beat measures that were 30–50 mmHg lower than the contralateral brachial measures. These sections were treated as missing values.

**Table 1:** Participant demographics.

Participant	NLI	AIS	TSI (weeks)	Age (years)	Height (cm)	Weight (kg)	Supine-Baseline BP (mmHg)	AFES intensity (mA)
**1**	C3	C	5	21	188	75	121/69	34.8
**2**	C3	C	7	78	178	99	131/61	48.8
**3**	C4	A	14	50	175	75	142/78	44.3
**4**	C4	A	3	47	185	95	118/69	47.5
**5**	C4	B	9	39	180	70	107/67	25.5
**6**	C4	B	6	29	185	65	123/83	60.0
**7**	C5	A	15	25	175	65	119/76	29.5
**8**	C5	D	9	76	194	120	121/82	54.8
**9**	C5	D	27	43	180	93	136/86	55.0
**10**	C7	B	11	49	175	80	145/86	N/A
**11**	T5	A	8	42	167	48	126/72	31.3

Characteristics of all participants, ordered by neurological level of injury (NLI). Age, time since injury (TSI), height and weight are reported at the time of enrolment. Blood pressure (BP) during the supine-baseline phase shows average systolic BP over diastolic BP. The abdominal functional electrical stimulation (AFES) intensity shown is the average across AFES and AFES-Binder conditions and left and right trunk for each participant. All participants were male. N/A indicates missing value due to participant withdrawal. AIS, American Spinal Injury Association (ASIA) Impairment Scale.

Table 2 presents the statistical summary of our BP findings.

**Table 2:** Summary of findings for significance of orthostatic drops and difference between upright BP and preceding supine-treatment BP across conditions.

	Control	Binder	AFES	AFES-Binder
**Orthostatic drop in BP**				
Minimum SBP	<0.001*	<0.001*	<0.001*	<0.001*
Minimum DBP	<0.001*	<0.001*	<0.001*	<0.001*
**BP maintenance**				
Mean MAP	0.007*	<0.001*	<0.001*	0.203
Mean SBP	No interaction effects	No interaction effects	No interaction effects	No interaction effects
Mean DBP	0.268	0.001*	0.001*	0.536

Values represent *p*-values derived from post-hoc statistical comparisons between supine phase with treatment condition applied (supine-treatment) and upright phases, for each condition. Orthostatic drop in BP = minimum upright BP relative to mean BP in the supine-treatment phase averaged across one-minute intervals. BP maintenance = mean upright BP averaged across one-minute intervals relative to mean BP in the supine-treatment phase averaged across one-minute intervals. There was no significant main interaction effect for SBP, hence post-hoc comparisons were not conducted. Where post-hoc comparisons were conducted, * indicates significance (*p* < 0.05). BP, blood pressure. SBP, systolic blood pressure. DBP, diastolic blood pressure. MAP, mean arterial pressure. AFES, abdominal functional electrical stimulation.

### Presence of orthostatic drops across conditions

There were significant orthostatic drops in BP of ≥20 mmHg in SBP (Table 3; *p* < 0.001) and ≥10 mmHg in DBP (*p* < 0.001) in all conditions. The minimum upright BP in the binder condition was lower than the AFES-Binder condition [SBP: (−11 mmHg [−21 to −2], p = 0.013); DBP: (−8 mmHg [−15 to −1], p = 0.027)]. Minimum upright SBP for the binder was lower than the AFES condition (−13 mmHg [−22 to −3], p = 0.004). No other comparisons between conditions in the upright position reached significance (Table 2).

**Table 3:** Mean change and orthostatic drops and their timing during the upright phase.

BP measure	Treatment Condition	Δ BP_mean_ upright (mmHg)	Δ BP_min_ upright (mmHg)	BP_min_ event <3 min (n)	BP_min_ event 3–10 min (n)	BP_min_ event >10 min (n)	BP_min_ time (minutes)	Total upright time (minutes)
**MAP**	Control	−5 [−11 to 1]	−21 [−28 to −15]	3	4	4	6.0 (2.0–12.5)	14.0 (9.5–15.5)
	Binder	−8 [−17 to 0]	−27 [−37 to −17]	4	5	1	3.0 (1.3–6.8)	15.0 (14.3–15.8)
	AFES	−6 [−11 to −1]	−20 [−25 to −14]	1	6	3	7.5 (3.5–13.0)	14.5 (11.5–16.0)
	AFES-Binder	−3 [−11 to 5]	−20 [−28 to −13]	4	4	2	4.5 (1.3–7.3)	14.5 (13.3–16.0)
**SBP**	Control	−8 [−17 to 1]	−32 [−42 to −22]	4	4	3	6.0 (0–10.5)	^x^
	Binder	−13 [−25 to −2]	−40 [−52 to −27]	6	1	3	2.0 (0.3–9.8)	^x^
	AFES	−8 [−16 to 0]	−29 [−38 to −19]	2	5	3	9.0 (4.3–12.3)	^x^
	AFES-Binder	−7 [−19 to 5]	−32 [−43 to −20]	2	5	3	6.5 (3.3–10.5)	^x^
**DBP**	Control	−2 [−6 to 1]	−18 [−22 to −13]	3	6	2	6.0 (2.0–8.0)	^x^
	Binder	−6 [−12 to 1]	−23 [−31 to −15]	4	5	1	3.0 (1.0–7.0)	^x^
	AFES	−4 [−8 to 0]	−17 [−22 to −11]	5	4	1	2.5 (2.0–7.5)	^x^
	AFES-Binder	0 [−6 to 7]	−16 [−22 to −11]	4	5	1	4.5 (1.0–8.0)	^x^

Mean differences [95% confidence interval] in blood pressure (BP) measures for the upright relative to supine positions. For orthostatic drops in BP, differences (Δ) were calculated as the minimum BP (BP_min_) when upright minus the mean BP (BP_mean_) when supine. For BP maintenance, differences were calculated as the change in BP_mean_ between postures. BP_min_ event shows the number of instances (n) in which BP_min_ occurred within each interval. BP_min_ time and total upright time are shown as medians (interquartile ranges). ^x^ indicates redundant measures. MAP, mean arterial pressure; SBP, systolic blood pressure; DBP, diastolic blood pressure. AFES, abdominal functional electrical stimulation.

### Main effects of condition and phase on mean blood pressure maintenance

**Fig. 2:**
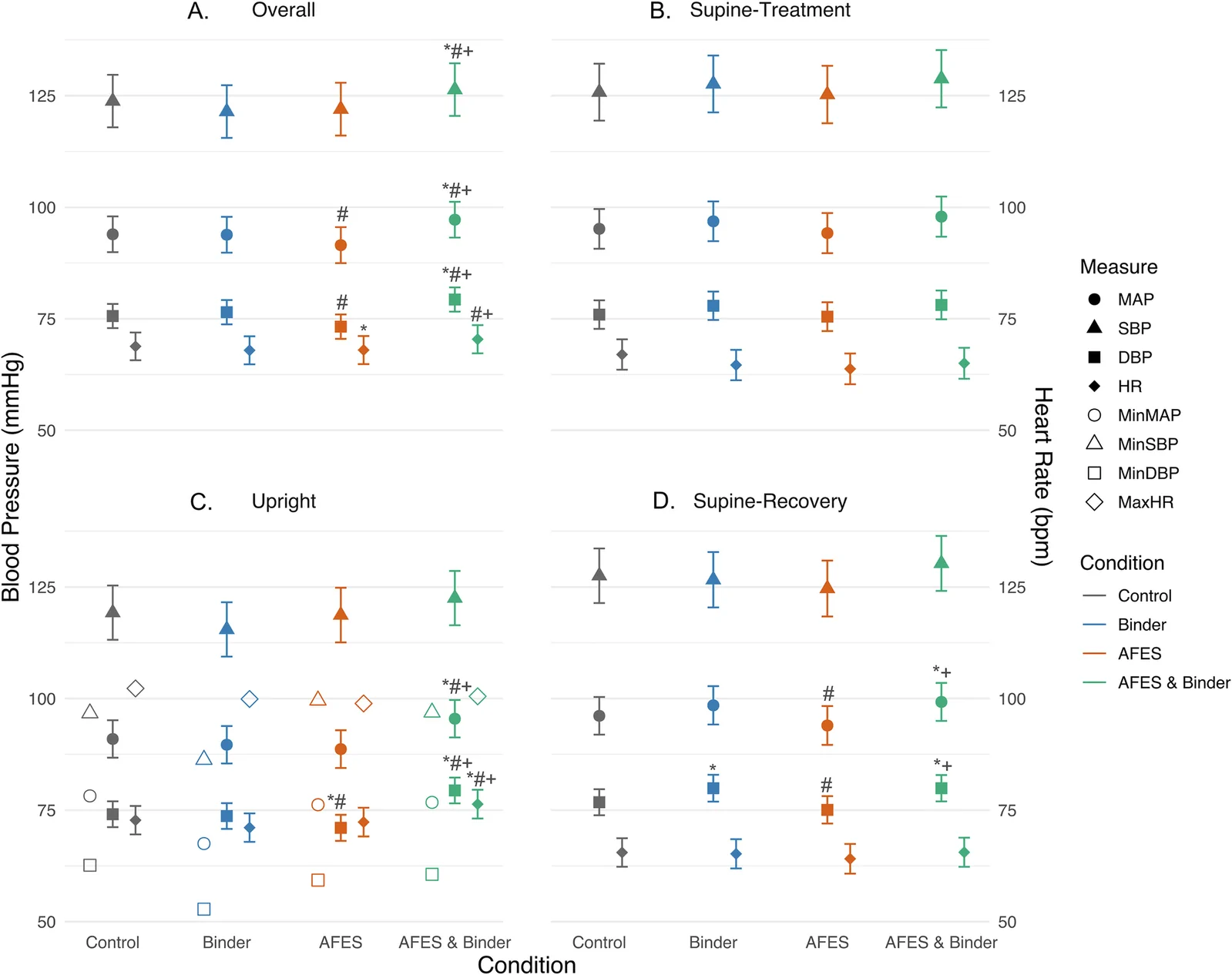
Comparison of blood pressure between conditions overall and within each phase. Estimated marginal means (±95% CI) for mean arterial pressure (MAP ●), systolic blood pressure (SBP ▲), diastolic blood pressure (DBP ■), and heart rate (HR ◆) for the four treatment conditions (no intervention control, binder, abdominal functional electrical stimulation (AFES), and AFES and binder combined). These are presented for each condition as **A** overall values derived across all phases, and during each phase **B** supine with treatment condition applied (supine-treatment), **C** upright with treatment condition applied and **D** returned to supine with treatment condition removed (supine-recovery) in 11 participants. Additionally, the means of minimum MAP (○), SBP (△), DBP (□) and maximum HR (◇) in the upright (**C**) phase. Refer to left y-axis for MAP, SBP and DBP, and to the right y-axis for HR. *indicates significant difference (*p* < 0.05) from no intervention (Control) condition; ^#^indicates significant difference (*p* < 0.05) from the Binder condition; ^+^indicates significant difference (*p* < 0.05) from the AFES condition.

There was a significant effect of condition on BP [MAP: (F_(3, 1090)_ = 14.97, *p* < 0.001); SBP: (F_(3, 1090)_ = 7.40, *p* < 0.001); DBP: (F_(3, 1092)_ = 20.83, *p* < 0.001)] (Fig. 2A). Mean overall BP was highest in the AFES-Binder condition (Table 4).

**Table 4:** Difference from no intervention control condition for blood pressure and heart rate across phases.

		Binder	AFES	AFES-Binder
**Overall**	MAP (mmHg)	1 [−1 to 3]	−2 [−4 to 0] ^#^	4 [1 to 6] *^#+^
	SBP (mmHg)	−1 [−4 to 2]	−1 [−4 to 1]	3 [0 to 6] *^#+^
	DBP (mmHg)	2 [0 to 3]	−2 [−4 to 0] ^#^	4 [2 to 5] *^#+^
	HR (bpm)	−1 [−3 to 0]	−2 [−3 to 0] *	1 [−1 to 2] ^#+^
**Supine Treatment**	MAP (mmHg)	2 [−3 to 6]	−1 [−5 to 3]	3 [−3 to 6]
	SBP (mmHg)	2 [−4 to 7]	−1 [−6 to 5]	3 [−3 to 9]
	DBP (mmHg)	2 [−2 to 6]	−1 [−4 to 3]	2 [−2 to 6]
	HR (bpm)	−2 [−6 to 1]	−3 [−7 to 0]	−2 [−6 to 1]
**Upright**	MAP (mmHg)	−1 [−4 to 2]	−2 [−5 to 1]	5 [2 to 7] *^#+^
	SBP (mmHg)	−4 [−7 to 0]	−1 [−4 to 3]	3 [0 to 7]
	DBP (mmHg)	0 [−3 to 2]	−3 [−6 to 0] *^#^	5 [3 to 8] *^#+^
	HR (bpm)	−2 [−4 to 1]	0 [3 to 2]	4 [1 to 6] *^#+^

Difference in estimated marginal means [95% CI] of mean mean arterial pressure (MAP), systolic blood pressure (SBP), diastolic blood pressure (DBP) and heart rate (HR) for the binder, AFES and AFES and binder combined (AFES-Binder) conditions to the no intervention control condition across all phases (overall), supine with condition applied (supine-treatment) phase and upright phase. * indicates statistically significant difference (*p* < 0.05) to the no intervention control condition; ^#^ indicates statistically significant difference (*p* < 0.05) to the binder condition; ^+^ indicates statistically significant difference (*p* < 0.05) to the AFES condition. AFES, abdominal functional electrical stimulation.

There was a significant effect of phase on BP [MAP: (F_(2, 1088)_ = 53.82, *p* < 0.001); SBP: (F_(2, 1088)_ = 12.11, *p* < 0.001); DBP: (F_(2, 1089)_ = 22.58, *p* < 0.001)], with lowest mean BP recorded in the upright phase [MAP: (91 mmHg [87 to 95]); SBP: (119 mmHg [113 to 125]); DBP: (75 mmHg [72 to 77])]. Mean SBP decreased significantly in the upright phase across all conditions (−8 mmHg [−10 to −6], *p* < 0.001).

### Interaction effects on mean blood pressure maintenance

There was an interaction effect between condition and phase for MAP and DBP, meaning that the changes across phases depended on the applied condition [MAP: (F_(6, 1087)_ = 2.15, p = 0.045); DBP: (F_(6, 1088)_ = 4.06, *p* < 0.001)]. However, there was no significant interaction effect on SBP (F_(6, 1087)_ = 2.21, p = 0.103). Therefore, only post-hoc comparisons for MAP and DBP are described below.

**Fig. 3:**
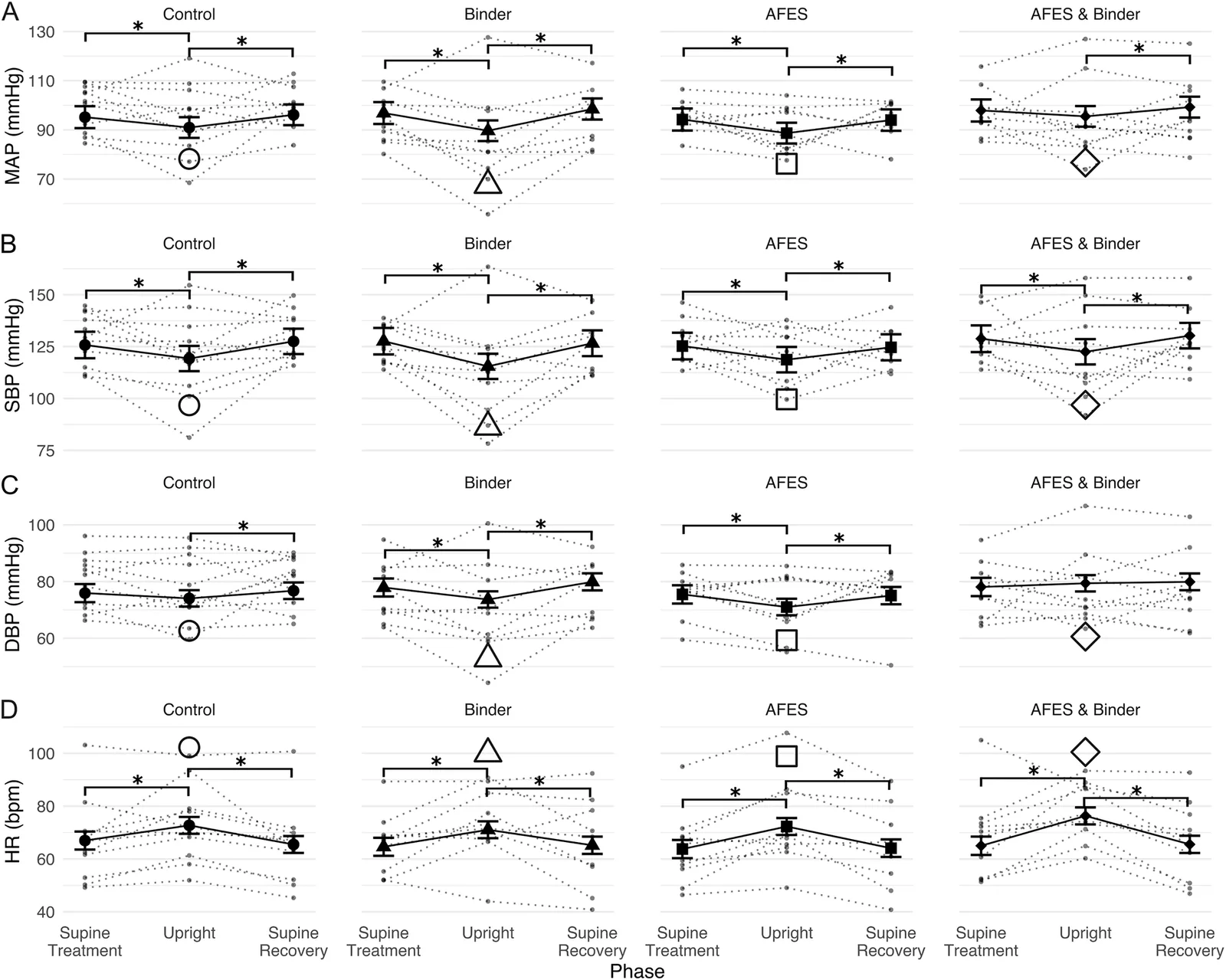
Blood pressure measurements for each condition across the supine condition, upright and supine recovery phases. Estimated marginal means (±95% confidence interval (CI)) for mean arterial pressure (MAP; **A**), systolic blood pressure (SBP; **B**), diastolic blood pressure (DBP; **C**) and heart rate (HR; **D**). The means of minimum (maximum for HR) values for each measure in no intervention (Control; ○), Binder (△), AFES (□) and AFES and binder (AFES & Binder; ◇) in the upright phase are also shown. *indicates significant difference between phases (*p* < 0.05). AFES, abdominal functional electrical stimulation.

For the AFES-Binder condition, there was no statistically significant difference in mean MAP between supine-treatment and upright phases (p = 0.203) (Fig. 3A). All other conditions showed significant decreases in mean MAP in the upright phase [Control: −4% (p = 0.007); Binder: −7% (*p* < 0.001); AFES: −6% (*p* < 0.001)]. Mean DBP did not change in the upright phase for the Control (p = 0.268) or AFES-Binder condition (p = 0.536) but dropped in the binder and AFES conditions [Binder: −5% (p = 0.001); AFES: −6% (p = 0.001)]. Overall, the AFES-Binder condition limited reductions in more BP outcomes compared to any other condition in the upright phase.

Mean MAP in the upright phase was significantly higher in the AFES-Binder condition compared to all other conditions [Control: (5 mmHg [95% CI, 2 to 7], *p* < 0.001); Binder: (6 mmHg [95% CI, 3 to 9], *p* < 0.001); AFES: (7 mmHg [95% CI, 4 to 10], *p* < 0.001)]. Similarly, mean upright DBP was highest in the AFES-Binder condition [Control: (5 mmHg [95% CI, 3 to 8], *p* < 0.001); Binder: (6 mmHg [95% CI, 3 to 8], *p* < 0.001); AFES: (8 mmHg [95% CI, 6 to 11], *p* < 0.001)] while it was lowest in the AFES condition [Control: (−3 mmHg [95% CI, −6 to −1], p = 0.011); Binder: (−3 mmHg [95% CI −5 to 0], p = 0.029)]. Overall, the AFES-Binder condition maintained higher upright BP compared to other conditions.

### Heart rate

There was a significant effect of condition (F_(3, 1046)_ = 5.68, p = 0.001), phase (F_(2, 1088)_ = 180.46, *p* < 0.001) and interaction (F_(6, 1087)_ = 3.84, p = 0.001) on mean HR. Mean HR was highest in the AFES-Binder condition during the upright phase [Control: (4 bpm [95% CI, 1 to 6], *p* < 0.001); Binder: (5 bpm [95% CI, 3 to 8], *p* < 0.001); AFES: (4 bpm [95% CI, 2 to 6], *p* < 0.001)].

### Presyncope scores

There was a significant effect of condition and phase on presyncope score. Presyncope score was higher in Control (0.5 [95% CI, 0.2 to 0.8]) compared to the AFES condition (−0.3 [95% CI, −0.1 to 0.5], p = 0.015). Control was the only condition in which the 95% CI did not cross zero [Binder: (0.2 [95% CI, −0.1 to 0.6]); AFES-Binder: (0.3 [95% CI, −0.1 to 0.6])].

## Discussion

When applied simultaneously, AFES and the binder maintained upright BP for individuals with subacute SCI more than any other condition (Table 2). Although their combined use improved BP maintenance during orthostatic challenges, it did not prevent OH. We hypothesised that the application of constant pressure [19] via the binder or intermittently via AFES, or the facilitation of venous return through AFES-induced muscle contraction [20], would improve BP outcomes. Each mechanism alone appears to be insufficient, but the simultaneous application of AFES and binder may maintain BP through a combination of both mechanisms. Maintenance of BP using AFES and an abdominal binder may support greater tolerance of the upright position in individuals with subacute SCI, which in turn could facilitate longer periods of upright functional activity.

### AFES + binder condition improved maintenance of upright BP but did not prevent orthostatic drops

This study is the first instance in which AFES and the binder were applied together to assess their combined effect on BP in people with SCI. AFES and the binder demonstrate potential to improve BP maintenance post-SCI through abdominal muscle contraction and compression [19, 20]. In the AFES-Binder condition of the present study, mean MAP did not decrease from the supine-treatment phase to the upright phase. Mean MAP dropped ~4–8% across the same phases in the other conditions. This suggests that AFES and the binder are complementary interventions and should be combined for maximal benefit.

The AFES-Binder condition may have enhanced muscle contraction, generating greater intra-abdominal and intra-thoracic pressures [21–24] compared to the AFES or Binder conditions. Application of the binder over the surface electrode pads may have decreased the distance between the skin and nerve, improving current delivery and the efficacy of AFES for muscle recruitment. This may have effectively increased the stimulation intensity, which has dose-dependent effects on intra-abdominal pressure [21]. In individuals with SCI, a ~ 50 mA increase in stimulation intensity corresponds to a ~ 5 cmH_2_O increase in intra-abdominal twitch pressure [21], which we expect to translate to a much larger increase in pressure during a 50 Hz train of AFES to reduce splanchnic pooling and increase BP [12].

The combined application of AFES and binder may have improved venous return, reflected in higher upright HR in the AFES-Binder condition compared to all other conditions. Although elevated HR is often associated with sympathetic activation as a compensatory autonomic response to falling BP, the modest levels of mean upright HR observed ( ~ 76 bpm, change of 11 bpm) may instead reflect a physiological adjustment to increased venous return to the heart [25].

### The binder alone did not improve maintenance of upright BP nor prevent orthostatic drops

While the abdominal binder has been studied for its effects on respiratory function and speech across different postures [24, 26], this is the first powered trial in SCI where the primary outcome was BP. Evidence of the binder’s effect on BP is mixed [26, 27]. Contrary to previous reports, where upright SBP with the binder was higher by ~7–8 mmHg [27] or did not significantly differ [26] from no binder, we found that orthostatic drops were greatest in the binder condition (Table 3). Differences in recruitment and protocols may explain our findings. First, our participants were selected based on signs or symptoms of OH, unlike previous studies [24, 26, 27], suggesting greater sympathetic impairment. Second, one of these studies assessed the effect of a binder on BP maintenance in a stable sitting position with non-continuous measures [27], whereas we incorporated a position change (supine to upright), which likely posed a stronger orthostatic challenge. We also recorded continuous beat-to-beat BP measurements, which are more sensitive to BP changes. A separate study [26] reported persistent SBP drops indicative of OH with the binder in 4 of 7 individuals who were symptomatic with OH. Therefore, while the binder may limit BP changes in some individuals, it appears insufficient to maintain the BP of individuals prone to OH with significant sympathetic impairments.

While our findings are novel, evidence exists that may explain why BP maintenance was worse in the Binder condition compared to Control. In athletes with SCI, the binder shifts the operating lung-volume range downward [28]. However, oxygen transport improves, likely as preserved respiratory muscle strength in athletes allows them to maintain inspiratory flow, which supports venous return [28]. In contrast, our non-athlete participants likely had reduced respiratory muscle strength and had not undergone the respiratory muscle training protocol that was found to improve BP maintenance during orthostatic stress [29]. It is possible that binder-induced abdominal pressure exceeded their inspiratory capacity, limiting flow and venous return. Our findings suggest the need for both pressure, exerted by the binder, and muscle pump, driven by AFES during expiration to improve BP maintenance in people with SCI who experience OH.

### AFES alone did not improve upright BP maintenance nor prevent orthostatic drops

AFES may increase BP by activating the abdominal muscles to increase intra-abdominal pressure and decrease splanchnic blood pooling [19, 20]. Consistent with this hypothesis, AFES paired with cough training acutely increased upright BP by ~32% in individuals with chronic SCI [13]. Electrical stimulation of the rectus abdominis, external oblique and erector spinae muscles also increased BP during 2 min of stimulation in wheelchair rugby athletes with SCI [30]. However, AFES alone in the present study failed to reduce orthostatic drops and averaged a lower upright DBP relative to the Control condition. Similarly, combined application of AFES with compression stockings did not prevent OH in another study [14].

The following factors may explain discrepancies across the studies. First, in Bojanic et al. [13], AFES was delivered with a cough vs quiet breathing in the current study. There is a transient increase in arterial diastolic and systolic pressures during a cough, without AFES, compared to quiet breathing [31]. Second, the average stimulation intensity in our study and Ratanapinunchai et al. [14], set to evoke a visible contraction, was 43.1 ± 13.3 mA (mean ± SD) and 48.6 ± 13.6 mA respectively, compared to 142.7 ± 10.0 mA [13] and 63.6 ± 19.9 mA [30]. However, the charge density, which refers to the amount of electrical charge delivered per unit electrode area, was similar in Bojanic et al. [13] and Ratanapinunchai et al. [14]. In contrast, the charge density in our study was approximately 50–60% of that reported in both studies. Therefore, both stimulation intensity and electrode size may determine AFES’ effects on BP, as sufficient intensity delivered through a large electrode appears more important than charge density alone. Third, studies which found increased BP with AFES did not assess BP throughout a position change (i.e. supine to upright). BP was only assessed in a stable upright position, with and without AFES [13, 30]. Meanwhile, with an orthostatic tilt-table test, AFES did not prevent OH [14]. This suggests that high-intensity AFES alone increases BP in a stable upright position, but lower intensity AFES is insufficient to prevent orthostatic drops during a sit-up or tilt test.

### Clinical implications

No condition prevented OH. However, the small but statistically significant difference in the maintenance of upright BP in the AFES-Binder condition may be clinically relevant to improve tolerance of upright positions in people with recent SCI. Direct clinical translation of this research is possible given our research was conducted in an acute spinal ward within a rehabilitation setting where binders and FES are used in practice.

### Limitations

Our study was not powered to identify the effect of time on the manifestation of OH, hence we could not compare BP behaviour across the different conditions across time after transition to the upright phase. OH typically occurs within 3 min after transition into the upright position [15], but can vary within the SCI population. Hence, a larger sample size would be needed to determine time-specific effects of each condition and the duration of the upright posture. Multiple outcomes were assessed. Concurrent evaluation of OH and BP maintenance using minimum and mean upright BP measures respectively, introduces a potential risk of Type I error due to multiple outcome analyses. The study also did not undergo prospective registration. Blinding was not feasible due to the nature of the interventions. We did not quantify internal pressures exerted by the interventions due to the setting nor potential diurnal variations (fatigue, hydration status, meal timing) that may influence BP [2]. Future investigations that include internal pressure assessments would help to clarify our results.

## Conclusions

While the abdominal binder and AFES, applied together or alone, did not prevent signs of OH in individuals with a subacute SCI, the AFES-Binder condition maintained higher overall upright BP to a small, but statistically significant magnitude compared to the other conditions. AFES alone may not generate sufficient intra-abdominal pressure to elevate BP, whereas the binder, without concurrent muscle contraction from AFES, may apply static pressure that restricts inspiratory flow and venous return. Improved upright BP maintenance observed with combined AFES and binder suggests that both muscle contraction and adequate intra-abdominal pressure are essential for effective circulatory support. Future studies should investigate higher AFES intensities to confirm whether improved BP with AFES and the binder reflects stimulation intensity, the binder, or both, and explore its effects alongside pharmacological strategies for personalised BP management. Overall, AFES with an abdominal binder could assist individuals with SCI to maintain upright BP and minimise the impacts of lowered upright BP in individuals with OH, particularly for those who do not respond to, or have contraindications for, other treatments.

## Data Availability

Additional data relating to this study are available from the corresponding author upon reasonable request.
